# Detection of Peptide-Based Nanoparticles in Blood Plasma by ELISA

**DOI:** 10.1371/journal.pone.0126136

**Published:** 2015-05-21

**Authors:** Gerard H. Bode, Karin E. Pickl, Maria Sanchez-Purrà, Berta Albaiges, Salvador Borrós, Andy J. G. Pötgens, Christoph Schmitz, Frank M. Sinner, Mario Losen, Harry W. M. Steinbusch, Hans-Georg Frank, Pilar Martinez-Martinez, European NanoBioPharmaceutics Research Initiative

**Affiliations:** 1 Department of Neuroscience, School for Mental Health and Neuroscience, Faculty of Health, Medicine and Life Sciences, Maastricht University, Maastricht, the Netherlands; 2 HEALTH—Institute for Biomedicine and Health Sciences, Joanneum Research Forschungsgesellschaft m.b.H, Graz, Austria; 3 Grup d'Enginyeria de Materials (GEMAT), Institut Químic de Sarrià, Universitat Ramon Llull, Barcelona, Spain; 4 AplaGen GmbH, Arnold-Sommerfeld-Ring 2, Baesweiler, Germany; 5 Department of Neuroanatomy, Ludwig-Maximilians-University of Munich, Munich, Germany; 6 Medical University of Graz, Department of Internal Medicine, Division of Endocrinology and Metabolism, Graz, Austria; University of Pécs Medical School, HUNGARY

## Abstract

**Aims:**

The aim of the current study was to develop a method to detect peptide-linked nanoparticles in blood plasma.

**Materials & Methods:**

A convenient enzyme linked immunosorbent assay (ELISA) was developed for the detection of peptides functionalized with biotin and fluorescein groups. As a proof of principle, polymerized pentafluorophenyl methacrylate nanoparticles linked to biotin-carboxyfluorescein labeled peptides were intravenously injected in Wistar rats. Serial blood plasma samples were analyzed by ELISA and by liquid chromatography mass spectrometry (LC/MS) technology.

**Results:**

The ELISA based method for the detection of FITC labeled peptides had a detection limit of 1 ng/mL. We were able to accurately measure peptides bound to pentafluorophenyl methacrylate nanoparticles in blood plasma of rats, and similar results were obtained by LC/MS.

**Conclusions:**

We detected FITC-labeled peptides on pentafluorophenyl methacrylate nanoparticles after injection *in vivo*. This method can be extended to detect nanoparticles with different chemical compositions.

## Introduction

The work presented here was developed by the FP6 EU biopharmaceutics platform, which aimed at the development of innovative multidisciplinary approaches for the design, synthesis and evaluation of molecular, nano- and micro-scale functionalities for targeted delivery of therapeutic peptides and proteins.

**Fig 1:**
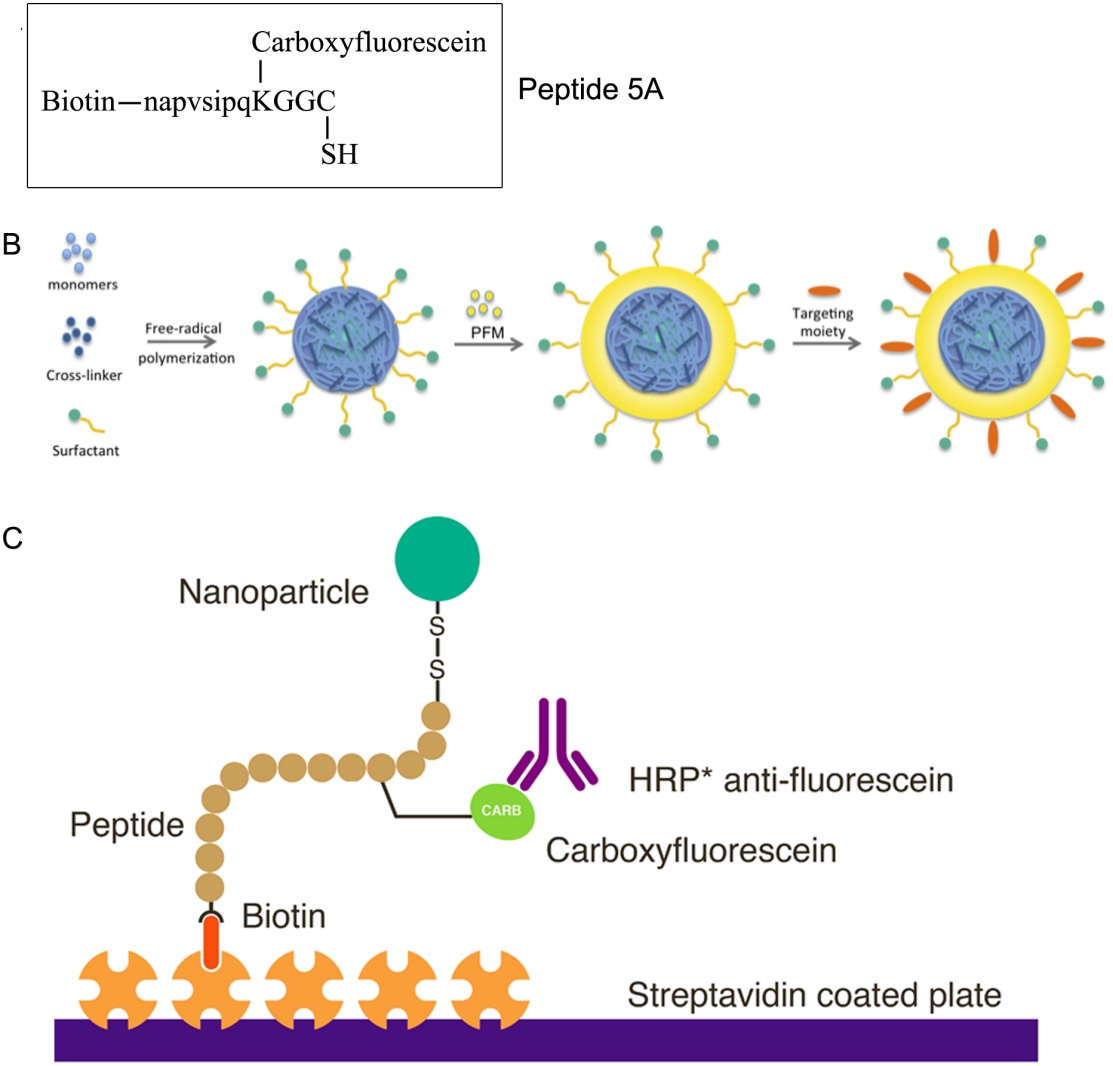
Schematic representation of the nanoparticle and ELISA design. Peptide 5A contains biotin and carboxyfluorescein, lower case letters denote D-amino acids. Nanoparticles were synthesized by a free-radical polymerization method in a microemulsion system. N-isopropylacrylamide, N,N-dimethylacrylamide and acrylic acid were used as monomers with methylenebisacrylamide as cross-linker. Pentafluorophenyl methacrylate (PFM) was added and the nanoparticles were adorned with peptide 5A (B). An ELISA was designed to detect nanoparticles in biological fluids using the biotin and carboxyfluorescein groups present on peptide 5A (C). Streptavidin coated plates were used to capture the peptides by binding the biotin group and bound peptides were detected with a HRP conjugated mAb anti-fluorescein.

The use of therapeutic peptides in neurodegenerative diseases is an active area of investigation. For example, the NAP peptide (NAPVSIPQ), derived from the activity-dependent neuroprotective protein (ADNP) [1], has shown efficacy in *in vitro* as well as *in vivo* models of neurodegenerative diseases [2, 3]. However, therapeutic peptides are known to have several limitations such as poor bioavailability, instability and short half-life [4]. A possible way to overcome these limitations is the use of nanoparticles as delivery method. Nanoparticles increase the bioavailability and efficacy of incorporated peptides by facilitating their transfer across biological membranes and protecting bound peptides against enzymatic degradation [5, 6]. Therapeutic nanoparticles are currently being developed for a wide variety of diseases such as cancer [7], cardiovascular disease [8–10] and neurodegenerative diseases [11, 12]. Although significant progress has been made towards organ-specific delivery of nanoparticles, a drawback is that they often do not reach their intended target tissue in the desired quantities due to filtering by the kidney, liver and spleen [7]. This can be improved by decorating the nanoparticles with functionalized targeting peptides that bind to receptors on the target tissue (Fig 1) [13]. Another challenge is the evaluation of the pharmacokinetics and biodistribution of the nanoparticles *in vivo*. In this respect LC-MS techniques can be used to measure peptides attached to nanoparticles. However, this approach requires specialized infrastructure. Therefore, it is useful to have reliable methods based on commonly used laboratory techniques, to be able to measure nanoparticles in biological fluids and tissues. In this manuscript we attached labeled reporter peptides to nanoparticles [6]. Subsequently, we used an ELISA-based method to detect the reporter peptide bound to the nanoparticles (Fig 1C), allowing quantification of these nanoparticles in blood plasma after injection *in vivo*. This ELISA enabled us to measure peptide bound to nanoparticles in blood plasma from 1 ng/mL. In parallel, LC/MS analysis of the same samples was performed to measure the plasma concentration over time of acrylamide based nanoparticles loaded with reporter peptides in rats.

## Materials and Methods

### Animals

All animal experiments were approved by the Maastricht University animal ethical committee and complied with Dutch law. Two month old male Wistar rats (Harlan, Ter Horst, the Netherlands) were used for the experiments. The animals were housed three per cage with *ad libitum* food and water and a 12:12 hour light:dark cyclus. A total of three animals was used in this study.

No anesthetics were used during blood sample collection. The animals were euthanized 24 hours after nanoparticle injection by intramuscular injection of ketamine (90 mg/kg) and xylazine (10 mg/kg) followed by transcardial perfusion with Tyrode’s buffer.

### Nanoparticles and peptides

N-isopropylacrylamide (NIPAAm, 97%), N,N-dimethylacrylamide (DMAAm, 99%), acrylic acid (AAc, 99%), methylenebisacrylamide (MBAAm, 99%), sodium dodecyl sulphate (SDS, ReagentPlus≥ 98.5%) and ammonium persulphate (98+%, A.C.S reagent) were all purchased from Sigma-Aldrich (St. Louis, MO) and used without further purification unless otherwise stated. Pentafluorophenyl methacrylate (PFM) was purchased from Apollo scientific, UK, cysteamine hydrochloride (purum ≥ 97.0%) and AlexaFluor 660 carboxylic acid succinimidyl ester from Invitrogen, US.

Nanoparticles were synthesized by a free-radical polymerization method in a microemulsion system [14], where the monomers used are N-isopropylacrylamide, N,N-dimethylacrylamide and acrylic acid, the cross-linker is methylenebisacrylamide, the surfactant, sodium dodecyl sulphate, and the initiator, ammonium persulphate.

For the synthesis of the nanoparticles a three-neck bottom-flask was charged with N-isopropylacrylamide (0.35 g, 3.09 mmol), N,N’-dimethylacrylamide (0.04 g, 0.4 mmol), acrylic acid (0.05 g, 0.69 mmol), methylenebisacrylamide (0.01 g, 0.06 mmol), sodium dodecyl sulphate (0.02 g, 0.07 mmol) and 117 mL of mili-Q water. The reaction mixture was heated at 70°C in a hot plate under reflux conditions, nitrogen atmosphere and stirring for four hours. After some minutes, when the reaction mixture was homogeneous and started to reflux, ammonium persulphate (0.036 g, 0.16 mmol) was added.

After 1 hour of reaction, pentafluorophenyl methacrylate (0.04 mmol) was added and the reaction mixture was further refluxed for 30 minutes. The obtained nanoparticles were further modified by the addition of the desired coating molecule. For thiolating, cysteamine hydrochloride (0.015 g, 0.13 mmol) was added, for labelling, Alexafluor 660 carboxylic acid succinimidyl ester (10 μg, 0.01 μmol). The peptide decoration was achieved via adding peptide 5A (1.055 mg, 0.39 mmol). Nanoparticle solutions were dialysed with dialysis membranes (nominal MWCO 6000–8000).

Nanoparticles size and zeta potential were determined using dynamic light scattering in a Nano ZS Nanosizer (Malvern Instruments Ltd., Malvern, UK) with a laser light wavelength of 632.8 nm and a scattering angle of 173 degrees. Temperature was set at 25°C. Nanoparticle solutions were measured without previous dilution. The mean diameter of the NPs was 174 nm and the zeta potential was -5.89 mV.

Peptide 5A (napvsipqKGGC, MW = 1754.0, monoisotopic mass = 1752.7; Fig 1A) was produced by conventional solid phase synthesis (AplaGen, Baesweiler, Germany). Peptide 5A+B (MW = 4694.2, monoisotopic mass = 4691.0) was produced by oxidizing peptide 5A with peptide 5B (Ac-CGGKTFFYGGCRGKRNNFKTEEY-COOH). Peptide 5D (isotopically labeled peptide 5A used as an internal standard for LC/MS, MW = 1760.0, monoisotopic mass = 1752.7) was prepared by labeling the two glycine amino acids with ^13^C_2_ and ^15^N_1_, resulting in a mass difference of +6 Da compared to peptide 5A. A schematic representation of peptide 5A, 5B and 5A+B is provided in Supporting Information S1 Fig

### Peptide 5A detection in blood plasma after intravenous injection of nanoparticles

The acrylamide based nanoparticles containing peptide 5A were injected in the lateral tail vein of 3 Wistar rats at a concentration of 17.25 mg of nanoparticle per kg of bodyweight, corresponding to 0.5 mg peptide 5A per kg of bodyweight. Blood samples were collected in lithium heparin plasma microtainer tubes (BD, Franklin Lakes, NJ). A small incision was made in the contralateral tail vein and the blood was collected directly into the tube. Immediately after collecting the blood, the tubes were manually inverted 8 times to ensure mixing with the anti-coagulant. Blood samples were stored on ice for up to 30 minutes before separating the plasma by centrifugation at 2000 g for 10 minutes. No hemolysis was observed.

Samples were collected from each animal immediately after injection (time point 0) and at 15, 30, 60, 120, 240, 480 minutes after injection. All plasma samples were stored at -80°C until further analysis.

### ELISA for the detection of peptide 5A

The plasma samples were diluted 1/500 in PBS, based on the range of the standard curve and the expected initial plasma concentrations. Streptavidin coated ELISA 96-well microplates (Steffens Biotechnische Analysen, Germany) were rinsed with 100 μl PBS per well and incubated with plasma samples in duplicate, for one hour at room temperature. After washing the wells three times with PBS, the carboxyfluorescein group present on the reporter peptide was detected by incubating with monoclonal (mAb) mouse-anti-fluorescein peroxidase-conjugated IgG (Dianova, Hamburg, Germany) diluted 1:100,000 in PBS with 0.1% BSA for one hour. Subsequently, the wells were washed three times with PBS and incubated with soluble high sensitivity TMB (SDT, Baesweiler, Germany) for 10 minutes in the dark. The enzymatic peroxidase reaction was stopped with 1 M HCl and absorbance at 450 nm was measured on a VICTOR *X*3 Multilabel Plate Reader (Perkin Elmer, Waltham, MA). All incubations were performed at room temperature on a plate shaker at 650 rpm. The wells were washed with PBS three times both after sample and antibody incubation. The TMB reaction was performed in the dark for 10 minutes. Negative controls consisted of incubating the wells with PBS instead of plasma samples and by using plasma without nanoparticles. The absorbance at 450 nm of the negative controls was considered as background and subtracted from the absorbance of the plasma samples.

Free peptide 5A was used as a positive control in the ELISA described above with a lower limit of detection of 1 ng/mL (data not shown). Nanoparticles containing a known concentration of peptide 5A, were used to generate a standard curve for the quantification of blood plasma samples. The standard curve data were fitted to a Hill curve using Graphpad Prism (Graphpad Software, La Jolla, CA) and the Hill equation was used to calculate the concentration of peptide 5A in blood plasma samples from the absorbance at 450 nm (See S1 Table).

### Liquid chromatography—mass spectrometry

#### Materials

Acetonitrile (>99.9%, for HPLC), water (for HPLC), formic acid (98–100%), acetic acid (>99%), trifluoroacetic acid (>99.5%), tris(2-carboxyethyl)phosphine (TCEP, C4706) and albumin from human serum (96–99%) were purchased from Sigma—Aldrich. Peptide 5A+B (the combined peptide 5A+B was produced by oxidizing peptide 5A with peptide 5B), peptide 5A and peptide 5D were synthesized by AplaGen, pooled rat EDTA plasma was received from Harlan. Ultracentrifugation devices (Amicon Ultra 4, 10 kDa MWCO) were obtained from Millipore (Billerica, MA).

#### Standards preparation

Peptide 5A standards were prepared by spiking rat plasma with peptide 5A+B and subsequently reducing the disulfide bond between peptide 5A and 5B, yielding free peptide 5A as depicted in Fig 1. The concentration range of the standards was 4.1–1674 ng/mL of peptide 5A. (Neither the peaks corresponding to the mass of peptide 5B nor the unreduced peptide 5A+B were analyzed, see LC-MS analysis section). In addition, two quality controls were prepared in rat plasma at concentrations of 83.7 and 837.1 ng/mL peptide 5A.

#### Sample preparation

Plasma samples for LC/MS were prepared by adding 300 μL of 10 mM TCEP in loading mobile phase mix to 30 μL of sample. 16 μL of internal standard (peptide 5D, isotopically labelled peptide 5A) was added and disulfide bonds were reduced by incubating at 60°C for 20 minutes. The samples were centrifuged at 8000 g for 5 minutes and subsequently filtered with 10 kDa MWCO filters at 500 g. The filtrate was transferred to autosampler vials and analyzed or frozen at -80°C until analysis.

Samples with expected concentrations higher than the upper limit of quantification were diluted with rat plasma prior to sample preparation.

#### LC/MS analysis

All LC/MS experiments were carried out on an Ultimate 3000 System (Dionex, LC Packings) coupled to a TSQ Quantum Ultra AM mass spectrometer (Thermo Finnigan). The system was controlled by Xcalibur Software 1.4. The chromatographic setup consisted of a capillary precolumn (Zorbax SBC3, 5 x 0.5 mm) in combination with a capillary analytical column (ACE 4, 250 x 0.3 mm). Sample loading was performed at 100 μL/min, elution at 4 μL/min. Chromatographic separation was carried out at 50°C. Twenty microliters were injected onto the precolumn using the μL-pickup mode. For sample loading onto the precolumn, acetonitrile:water solutions containing 1% acetic acid and 0.05% TFA were used (A: 5:94:1:0.05 ACN:H_2_O:AA:TFA, B: 95:4:1:0.05 ACN:H_2_O:AA:TFA, gradient: 0–5 minutes 10% B, 5–12 minutes 10 to 70% B, 12–14 minutes 70–100% B, 14–19.9 minutes 100% B, 19.9–20 minutes 100 to 10% B, 20–30 minutes 10% B). Elution was performed using a water: acetonitrile gradient containing 0.1% formic acid (A: 5:95:0.1:ACN:H_2_O:FA, B: 95:5:0.1:ACN:H_2_O:FA, gradient: 0–5 minutes 15% B, 5–10 minutes 15 to 30% B, 10–13 minutes 30% B, 13–14 minutes 30 to 100% B, 14–19 minutes 100% B, 19–19.1 minutes 100 to 15% B, 19.1–30 minutes 15% B). Valve switching times were 5 minutes and 12 minutes.

MS detection was performed in positive electrospray ionization (ESI) with a nanoESI source using the selected reaction monitoring (SRM) mode. The double-protonated molecule ion of the whole intact peptide was chosen as the parent ion, the three most intensive fragment ions were chosen as daughter ions. (Peptide 5A: m/z 877.4 → (341 + 412 + 946), peptide 5D: m/z 880.5 → (412.2 + 341.1 + 952.4). As mentioned in the standards preparation section, the peaks corresponding to peptide 5B and the unreduced peptide 5A+B were not analyzed. Quantification (see S2 Table for details) was carried out using the sum of all three SRM chromatograms in order to obtain good sensitivity.

## Results

### ELISA for the detection of carboxyfluorescein-labeled peptide 5A

**Fig 2:**
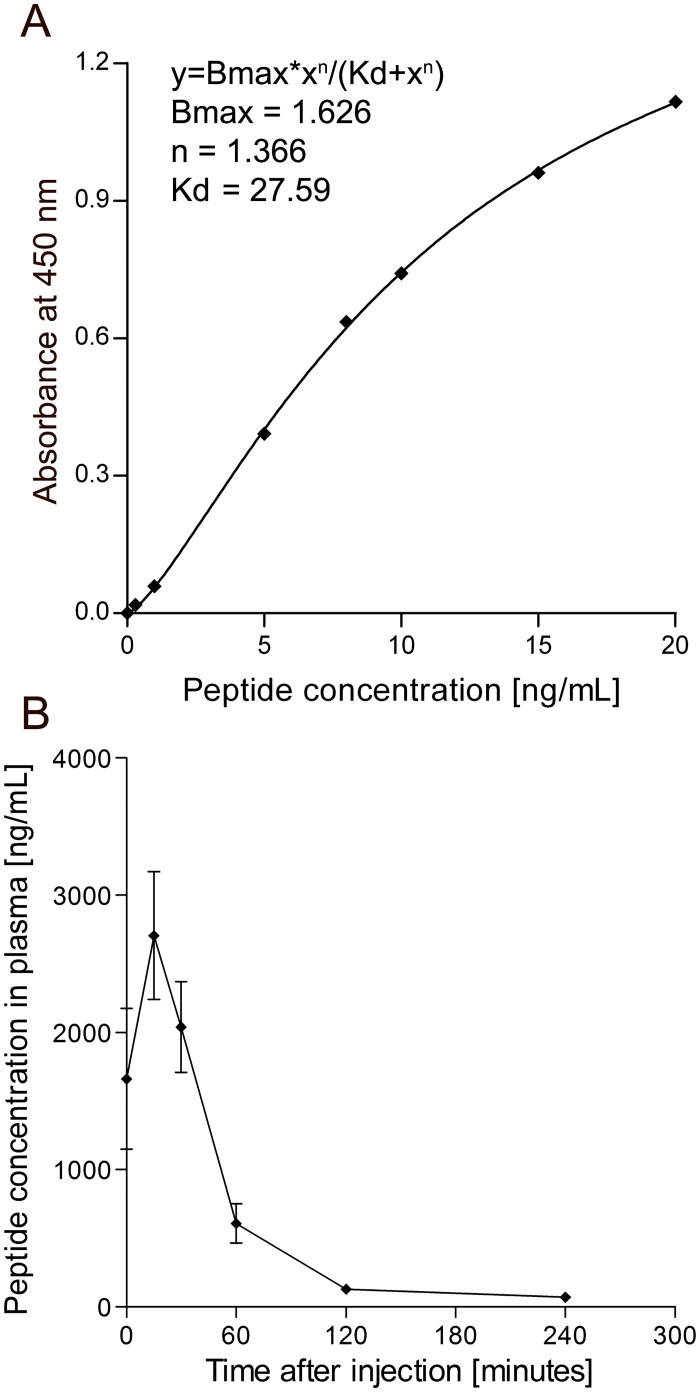
Quantification of peptide 5A in blood plasma by ELISA. A standard curve was generated using nanoparticles decorated with peptide 5A in a concentration range of 0.3 to 20 ng/mL peptide (A). Nanoparticles containing peptide 5A were injected into the lateral tail vein of male Wistar rats (n = 3). Plasma was prepared from venous blood samples collected at different time points after injection and peptide 5A concentration was measured by ELISA (B). Data shown are the mean ± S.E.M. peptide concentration in the plasma of three individual animals. The standard curve was generated using the Hill equation.

We developed an ELISA to detect carboxyfluorescein-labeled peptide 5A (see Fig 1C for schematic representation). In addition to the carboxyfluorescein, peptide 5A contains a biotin molecule. The peptide was selectively bound to the streptavidin coated plate via the biotin residue; subsequently a mAb against the carboxyfluorescein was used to detect the bound peptide. To test the sensitivity of the ELISA a dilution range of acrylamide-based nanoparticles with an equivalent of 0 to 20 ng/mL of carboxyfluorescein-labeled peptide 5A was added to the ELISA plate. The particles were detected with high sensitivity and the data were fitted to a standard curve using the Hill equation, with a Hill coefficient of 1.366 and a K_d_ of 27.59 ng/mL (Fig 2A).

### Detection of peptide 5A nanoparticles in blood plasma by ELISA

After confirming the specificity and sensitivity of the ELISA *in vitro*, we intravenously injected Wistar rats with the acrylamide based nanoparticle formulation containing the 5A reporter peptide described above. Blood samples were collected immediately and up to 24 hours after injection (data not shown). Plasma was prepared from venous blood and diluted 1/500 in PBS. The same ELISA as described above was used to measure the concentration of peptide 5A in the plasma samples. At time point 0 the mean ± S.E.M. of peptide 5A plasma concentration in 3 animals was 1661 ± 514 ng/mL, with the highest mean ± S.E.M. concentration of 2706 ± 466 ng/mL measured 15 minutes after injection (Fig 2B). We were able to detect the peptide up to 2 hours after injection (Fig 2B; absorbance data can be found in S3 Table). The results were reproducible when repeated several times. We noted that dilution of samples in normal rat plasma, rather than PBS, increased the maximum OD, while it had very little effect on the background (data not shown).

### Liquid chromatography—mass spectrometry quantification of peptide 5A in blood plasma of nanoparticle injected rats

**Fig 3:**
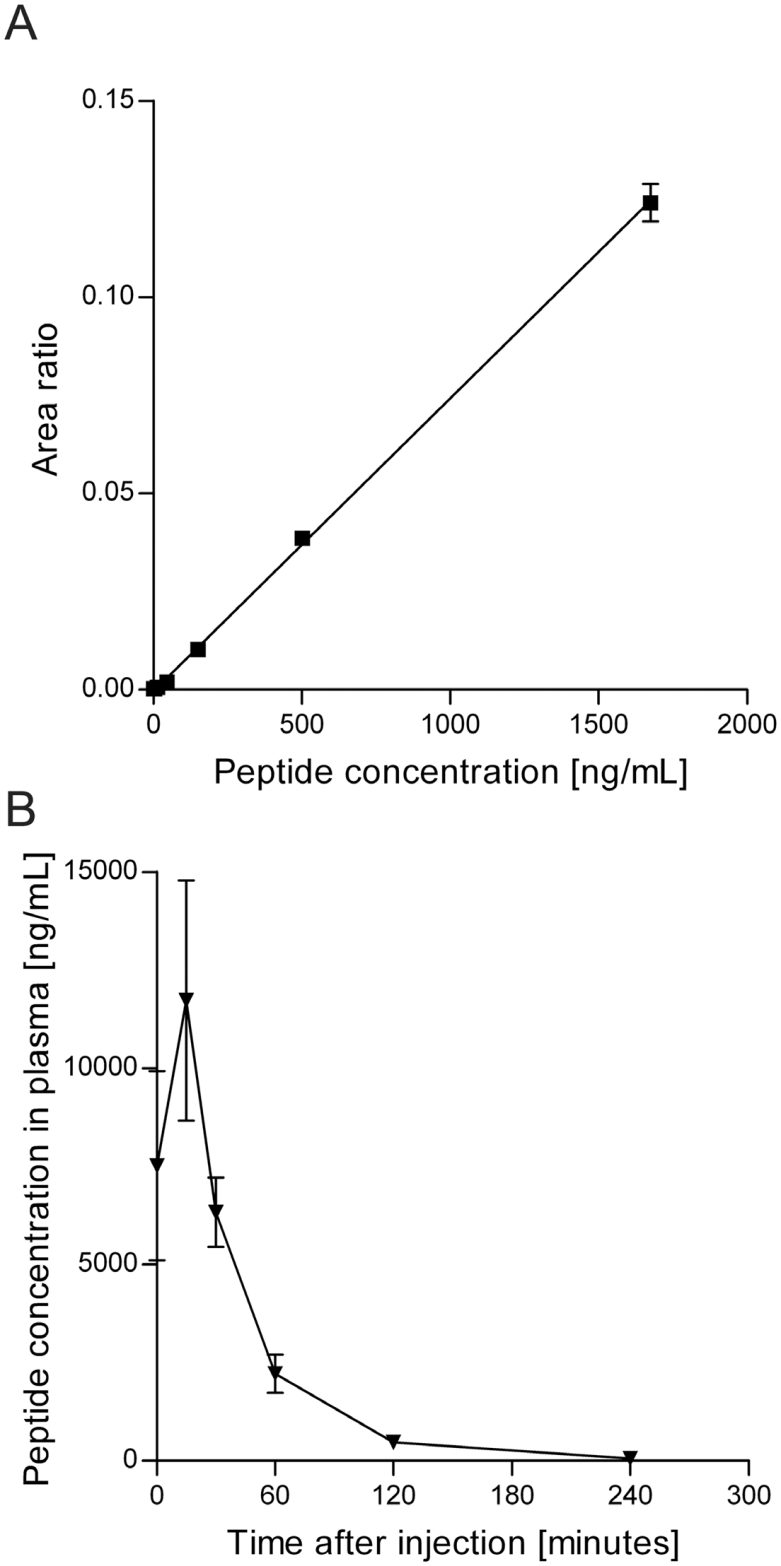
Quantification of peptide 5A in blood plasma by LC/MS. A standard curve for the quantification of peptide by LC/MS was generated using peptide 5A in a concentration range of 4.1 to 1674 ng/mL peptide (A). Blood plasma samples of animals injected with peptide 5A decorated nanoparticles were reduced and free peptide was quantified by LC/MS (B). Data shown are the mean ± S.E.M. peptide concentration in the plasma of three individual animals.

**Fig 4:**
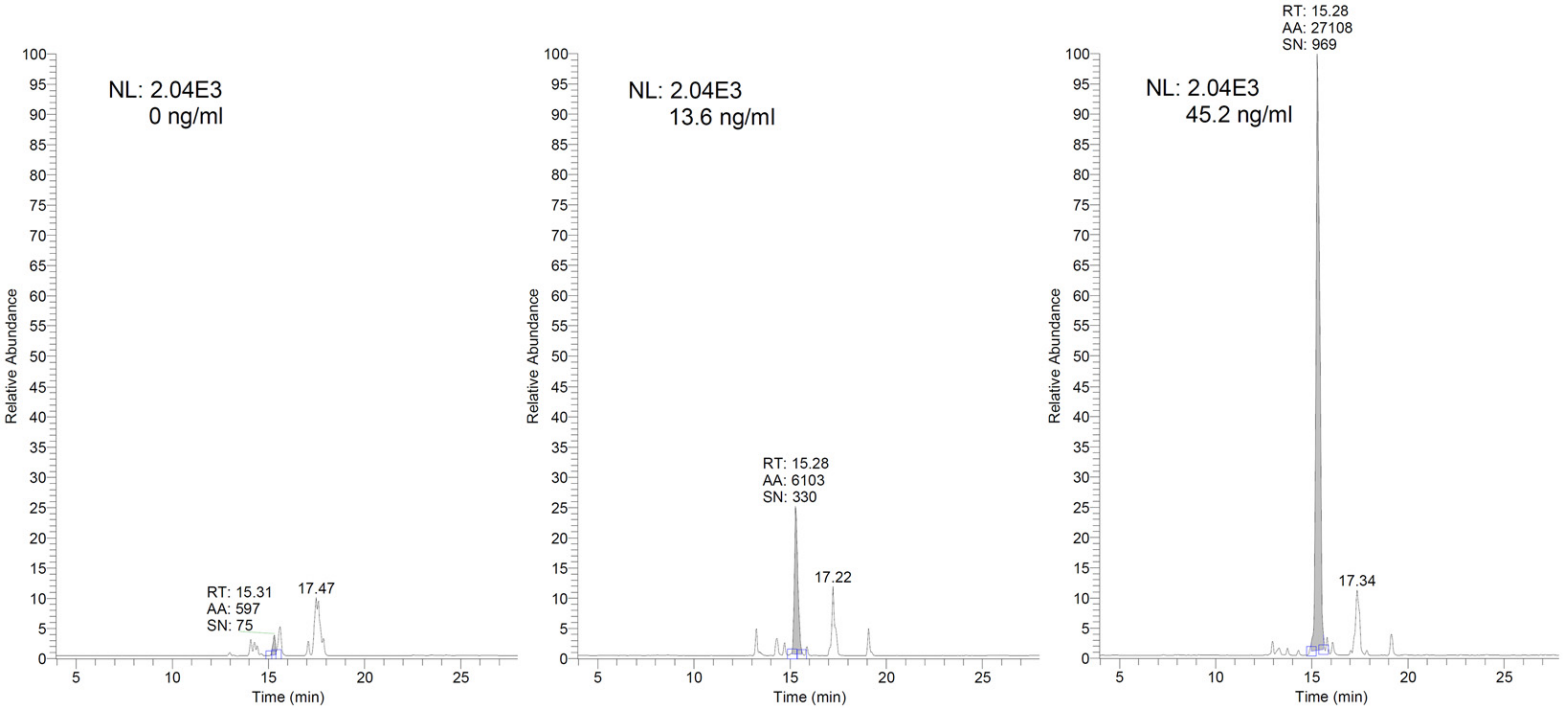
LC/MS chromatograms comparing different concentrations of peptide 5A in blood plasma matrix. Peptide 5A peaks in plasma matrix spiked with 0, 13.6 and 45.2 ng/mL (SRM trace: 877.7 → (412.2 + 341.1 + 946.4).

Peptide 5A used for generating the LC/MS standard curve was obtained by diluting the combined 5A+B peptide in plasma and subsequently reducing the disulfide bond between peptide 5A and peptide 5B. The LC/MS method showed good linearity in the range from 4.1 up to 1674 ng/mL (Fig 3A) with an expected lower limit of quantification of 13.5 ng/mL (S/N at 13.5 ng/mL was 330, Fig 4). Quality controls at 83.7 and 837 ng/mL measured in parallel with the *in vivo* study samples showed accuracies ranging between -7% and +7%.

LC/MS quantification of peptide 5A in blood plasma showed a similar pharmacokinetic profile as the ELISA based quantification. Interestingly, the peptide concentrations measured by LC/MS were approximately a factor 4 higher compared to the ELISA measurements. Immediately after injection a mean ± S.E.M. concentration of 7515 ± 2403 ng/mL was measured (Fig 3B; Peak area ratios can be found in S4 Table). As with the ELISA measurements, the highest concentration of 11724 ± 3062 ng/mL was found at the 15 minutes post-injection time point.

## Discussion

We have developed a convenient ELISA method for detecting a peptide conjugated to a nanoparticle, *in vivo* in rat plasma. By using a HRP conjugated antibody against FITC instead of measuring fluorescence directly, high sensitivity can be attained without the need for a spectrofluorometer. The ELISA proved to be highly sensitive, detecting the peptide 5A with a detection limit of 1 ng/mL. In addition, the ELISA detected peptide 5A in blood plasma after injection of nanoparticles decorated with peptide 5A in the lateral tail vein of Wistar rats. The blood plasma profile showed a maximum concentration of peptide in plasma 15 minutes after injection. The peptide was detectable in blood plasma up to 2 hours after injection (Fig 2B). The LC/MS analysis of the same samples also showed the highest peptide concentration after 15 minutes (Fig 3B). However, the measured peptide concentrations in plasma were higher by LC/MS than those measured by ELISA. There are several potential reasons for this difference. For example, the ELISA detects peptide attached to the nanoparticle and it is possible that not all of the peptide was accessible due to steric hindrance. In contrast, the LC/MS method detects the peptide that is released from the nanoparticle by reduction of the disulfide bond. Another factor that could account for the differences between the ELISA and LC-MS measurements are the different methods which are required for the preparation of the standards/samples.

In conclusion, we demonstrated that, the labeling of acrylamide nanoparticles with peptides containing functional groups such as biotin and carboxyfluorescein allows for detection of these nanoparticles by ELISA in blood plasma. This suggests that this technique is useful for the study of pharmacokinetics of nanoparticles *in vivo* and the development of therapeutic nano delivery systems. Finally, this method can be easily adapted for labeling of other nanoparticles with different chemical compositions and the detection of nanoparticles in other biological fluids and tissue, such as cerebrospinal fluid, brain and liver (manuscript in preparation).

## Supporting Information

S1 Fig Schematic representation of the structure of peptide 5A, peptide 5B and peptide 5A+B. (TIF)

S1 Table Absorbance data of the ELISA standards. The raw absorbance values of the duplicates at 450 nm and the average values are shown. A Hill equation was used to fit the data to a curve, which yielded the formula Concentration = (27.59*Absorbance/(1.626-Absorbance))^(1/1.366). This formula was subsequently used to calculate the peptide 5A concentrations of the samples.
(DOCX)

S2 Table Concentrations and area ratios of the LC/MS standards. The formula derived from the standard curve to calculate the concentrations of the samples was: Concentration = (Area ratio+4.32257·10^–4^)/7.46704·10^–5^
(DOCX)

S3 Table ELISA absorbance data of the plasma samples shown in Fig 2. Blank subtracted absorbance at 450 nm of 1 to 500 diluted plasma samples. Values shown are the average of duplicates.
(DOCX)

S4 Table Raw data of LC/MS analysis. Peak area ratios and dilution factors of the plasma samples shown in Fig 3. Values shown are the average of duplicates.
(DOCX)
