# Supplementary figures and images for: Detection of Peptide-Based Nanoparticles in Blood Plasma by ELISA
Source: PLoS One. 2015 May 21;10(5):e0126136. doi: 10.1371/journal.pone.0126136 (PMC4440766; doi:10.1371/journal.pone.0126136)

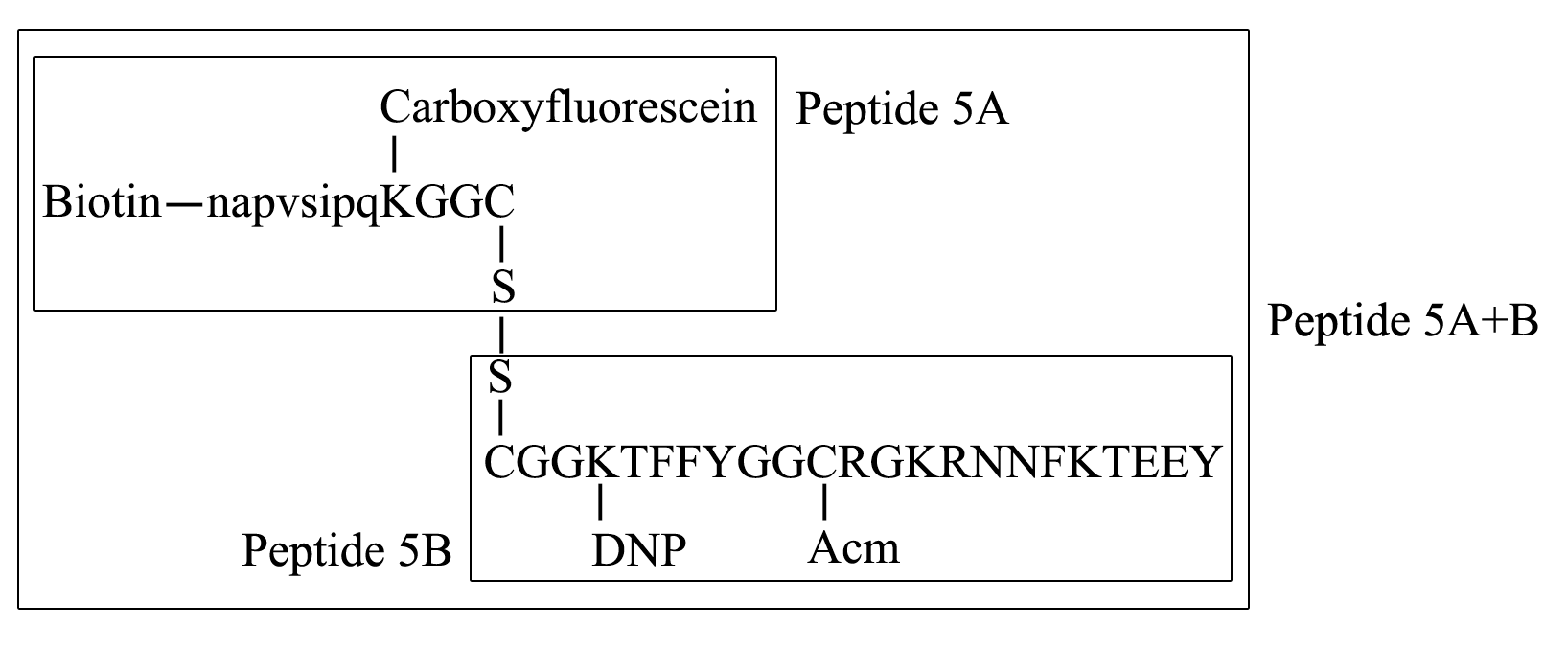

Supplement: S1 Fig — (TIF) [file pone.0126136.s001.tif]
