# Supplementary material for: Detection of Peptide-Based Nanoparticles in Blood Plasma by ELISA
Source: PLoS One. 2015 May 21;10(5):e0126136. doi: 10.1371/journal.pone.0126136 (PMC4440766; doi:10.1371/journal.pone.0126136)
Supplement: S1 Table — The raw absorbance values of the duplicates at 450 nm and the average values are shown. A Hill equation was used to fit the data to a curve, which yielded the formula Concentration = (27.59*Absorbance/(1.626-Absorbance))^(1/1.366). This formula was subsequently used to calculate the peptide 5A concentrations of the samples. (DOCX) [file pone.0126136.s002.docx]

S1 Table:

| Concentration of peptide 5A [ng/mL] | Absorbance at 450 nm (duplicates) | | Absorbance at 450 nm (average) | Blank subtracted absorbance at 450 nm | Calculated concentrations |
| --- | --- | --- | --- | --- | --- |
| 0 | 0.0537024 | 0.055911 | 0.055 |  |  |
| 0.3 | 0.0730453 | 0.072266 | 0.073 | 0.018 | 0.42 |
| 1 | 0.122231 | 0.105595 | 0.114 | 0.059 | 1.03 |
| 5 | 0.458871 | 0.433778 | 0.446 | 0.392 | 4.89 |
| 8 | 0.683941 | 0.696435 | 0.690 | 0.635 | 8.19 |
| 10 | 0.798588 | 0.793714 | 0.796 | 0.741 | 9.97 |
| 15 | 1.02919 | 1.00217 | 1.016 | 0.961 | 14.85 |
| 20 | 1.2159 | 1.12597 | 1.171 | 1.116 | 20.13 |
|  |  |  |  |  |  |
| 0 (plasma) |  |  | 0.058 |  |  |
