# Supplementary material for: Detection of Peptide-Based Nanoparticles in Blood Plasma by ELISA
Source: PLoS One. 2015 May 21;10(5):e0126136. doi: 10.1371/journal.pone.0126136 (PMC4440766; doi:10.1371/journal.pone.0126136)
Supplement: S2 Table — The formula derived from the standard curve to calculate the concentrations of the samples was: Concentration = (Area ratio+4.32257·10–4)/7.46704·10–5 (DOCX) [file pone.0126136.s003.docx]

S2 Table:

| Concentration [ng/mL] | Area ratio (triplicates) | | | Average |
| --- | --- | --- | --- | --- |
| 0 | Peak not found | Peak not found | 0.00022 | 0.000222 |
| 4.1 | 0.00022 | 0.00023 | Peak not found | 0.000225 |
| 13.6 | 0.00033 | 0.00074 | 0.00070 | 0.000589 |
| 45.2 | 0.00200 | 0.00068 | 0.00314 | 0.001939 |
| 150.7 | 0.01019 | 0.00989 | 0.01063 | 0.010237 |
| 502.3 | 0.03668 | 0.04084 | 0.03821 | 0.038576 |
| 1674.2 | 0.11930 | 0.12875 | not injected | 0.124024 |
