# Supplementary material for: Detection of Peptide-Based Nanoparticles in Blood Plasma by ELISA
Source: PLoS One. 2015 May 21;10(5):e0126136. doi: 10.1371/journal.pone.0126136 (PMC4440766; doi:10.1371/journal.pone.0126136)
Supplement: S3 Table — Blank subtracted absorbance at 450 nm of 1 to 500 diluted plasma samples. Values shown are the average of duplicates. (DOCX) [file pone.0126136.s004.docx]

S3 Table:

|  | Blank subtracted absorbance at 450 nm | | |
| --- | --- | --- | --- |
| Time after injection [minutes] | Animal 1 | Animal 2 | Animal 3 |
| 0 | 0.374 | 0.081 | 0.314 |
| 15 | 0.407 | 0.315 | 0.566 |
| 30 | 0.384 | 0.205 | 0.374 |
| 60 | 0.093 | 0.032 | 0.101 |
| 120 | 0.010 | 0.008 | 0.009 |
| 240 | 0.004 | 0.005 | 0.003 |
