# Supplementary material for: Detection of Peptide-Based Nanoparticles in Blood Plasma by ELISA
Source: PLoS One. 2015 May 21;10(5):e0126136. doi: 10.1371/journal.pone.0126136 (PMC4440766; doi:10.1371/journal.pone.0126136)
Supplement: S4 Table — Peak area ratios and dilution factors of the plasma samples shown in Fig 3. Values shown are the average of duplicates. (DOCX) [file pone.0126136.s005.docx]

S4 Table:

|  | Animal 1 | | Animal 2 | | Animal 3 | |
| --- | --- | --- | --- | --- | --- | --- |
| Time after injection [minutes] | peak area ratio | dilution factor | peak area ratio | dilution factor | peak area ratio | dilution factor |
| 0 | 0.07512 | 10 | 0.01983 | 10 | 0.07209 | 10 |
| 15 | 0.07085 | 10 | 0.58618 | 1 | 0.13226 | 10 |
| 30 | 0.05131 | 10 | 0.34220 | 1 | 0.05534 | 10 |
| 60 | 0.23438 | 1 | 0.11027 | 1 | 0.11304 | 1.33 |
| 120 | 0.02514 | 1.33 | 0.03947 | 1 | 0.03057 | 1 |
| 240 | 0.00255 | 1 | 0.00511 | 1 | 0.00312 | 1 |
